# A deep learning-driven low-power, accurate, and portable platform for rapid detection of COVID-19 using reverse-transcription loop-mediated isothermal amplification

**DOI:** 10.1038/s41598-022-07954-2

**Published:** 2022-03-08

**Authors:** Waqas Waheed, Sueda Saylan, Taimur Hassan, Hussain Kannout, Habiba Alsafar, Anas Alazzam

**Affiliations:** 1 Department of Mechanical Engineering, Khalifa University, Abu Dhabi, UAE; 2 System on Chip Center (SOCC), Khalifa University, Abu Dhabi, UAE; 3 Department of Electrical Engineering and Computer Science, Khalifa University, Abu Dhabi, UAE; 4 Center for Cyber-Physical Systems (C2PS), EECS Department, Khalifa University, Abu Dhabi, UAE; 5 Center for Biotechnology (BTC), Khalifa University, Abu Dhabi, UAE; 6 College of Medicine and Health Sciences, Khalifa University, Abu Dhabi, UAE

**Keywords:** Infectious diseases, Reverse transcription polymerase chain reaction

## Abstract

This paper presents a deep learning-driven portable, accurate, low-cost, and easy-to-use device to perform Reverse-Transcription Loop-Mediated Isothermal Amplification (RT-LAMP) to facilitate rapid detection of COVID-19. The 3D-printed device—powered using only a 5 Volt AC-DC adapter—can perform 16 simultaneous RT-LAMP reactions and can be used multiple times. Moreover, the experimental protocol is devised to obviate the need for separate, expensive equipment for RNA extraction in addition to eliminating sample evaporation. The entire process from sample preparation to the qualitative assessment of the LAMP amplification takes only 45 min (10 min for pre-heating and 35 min for RT-LAMP reactions). The completion of the amplification reaction yields a fuchsia color for the negative samples and either a yellow or orange color for the positive samples, based on a pH indicator dye. The device is coupled with a novel deep learning system that automatically analyzes the amplification results and pays attention to the pH indicator dye to screen the COVID-19 subjects. The proposed device has been rigorously tested on 250 RT-LAMP clinical samples, where it achieved an overall specificity and sensitivity of 0.9666 and 0.9722, respectively with a recall of 0.9892 for C_t_ < 30. Also, the proposed system can be widely used as an accurate, sensitive, rapid, and portable tool to detect COVID–19 in settings where access to a lab is difficult, or the results are urgently required.

## Introduction

In December 2019, a novel coronavirus emerged in Wuhan, China, later dubbed “severe acute respiratory syndrome coronavirus-2” (SARS-CoV-2). The SARS-CoV-2 is responsible for causing an acute respiratory disease called the coronavirus disease 2019 (COVID-19). After wreaking havoc in Wuhan, the disease rapidly spread across the globe within no time and was ultimately classified as a pandemic by World Health Organization (WHO) on March 11, 2020^1^. The disease has impacted our daily lives in unprecedented ways and has put the whole world on a standstill inflicting heavy economic and emotional losses in addition to bringing illness, suffering, and death. According to the WHO, the number of positive cases by Sept. 16, 2021, has amounted to greater than 226 million people with more than 4.6 million fatalities globally^2^. The major symptoms of COVID-19 are strikingly similar to the previous respiratory diseases, such as SARS and MERS. Nevertheless, COVID-19, initially thought to damage only the lungs, exceeds way beyond the lungs and affects other organs such as the heart, brain, kidneys, and the endocrine system^3^. Moreover, COVID-19 is estimated to be far more contagious compared to influenza A, SARS, and MERS^4^. A recent report by the US Centers for Disease Control and Prevention (CDC) estimates that nearly 50% of the new infections are transmitted to others from infected humans before they exhibit any signs of the disease^5^. The report further states that almost 40% of the infected patients never show any symptoms (asymptomatic)^5^, making the detection of COVID-19 an extremely daunting task.

### Related works

The medical practitioners are currently employing multiple methods to diagnose the COVID-19 disease. One of the most popular and accurate methods is the Reverse Transcription quantitative Polymerase Chain Reaction (RT-qPCR)^6^, which the WHO and the CDC declared as the gold standard for the detection of SARS CoV-2^7,8^. However, the method has its limitations. For instance, it requires specialized, bulky equipment and a highly-skilled workforce. Moreover, it requires a robust control and optimization of the heating/cooling modules operating at different temperatures; any inconsistencies in the heating/cooling temperatures or transition times during the PCR cycles could result in nonspecific or even no amplification^9,10^. Furthermore, the detection of the amplification poses additional challenges. It requires specialized equipment—such as electrophoresis or fluorescence-based equipment—thus adding to the complexity and the system's cost and overall process time. These challenges make it difficult to transport the PCR technique outside a specialized lab and utilize it in a point-of-care (POC) setting. On the other hand, the POC-based devices ay assist in the screening of large masses outside of laboratories. These devices can minimize the number of unnecessary visits to the labs/hospitals, reducing not just the burden of healthcare workers but also the risks of virus spreading. Furthermore, the rapid POC-based molecular tests would allow governments to conduct more diagnostic tests in parallel. Thus, more asymptomatic patients are likely to be detected, allowing for more efficient control in the fight against pandemic spread.

Other detection methods—called serology tests—detect antibodies or antigens associated with SARS-CoV-2. The serology tests are rapid, easy to use, cheaper, less complicated, and allow POC operation^11,12^. However, the antibody tests do not confirm the active state of infection in a patient, as they rely on the antibodies that the patient's immune system produces in response to SARS-CoV-2^12,13^. Moreover, these tests suffer from low accuracy, low sensitivity, and a high number of false-positive/negative results^13^. Therefore, there is a high demand for introducing alternative accurate POC-based detection methods. In this respect, a class of techniques called “isothermal amplification” appears to be a promising alternative. In contrast to the PCR method, the isothermal techniques require only a single temperature to carry out the nucleic acid amplification. Furthermore, the temperature requirement in these techniques ranges typically from 37 °C to 65 °C, much less than that used in the PCR denaturation step (~ 90 °C–95 °C). This greatly simplifies the heating requirements of the isothermal amplification-based systems, as there is no need of thermocyclers anymore. In addition, it makes this technique to be easily employed in portable devices that can be used in POC settings. Some of the examples include the helicase-dependent amplification (HDA)^14^, strand-displacement amplification (SDA)^15^, nucleic acid sequence-based amplification (NASBA)^16^, rolling circle amplification (RCA)^17^, and loop-mediated isothermal amplification (LAMP)^18^.

Among the isothermal amplification-based diagnosis tests, LAMP has emerged as an attractive amplification method for the POC applications because of its simplicity, high tolerance against inhibitors, and ability to amplify minimally processed or even raw samples^19–21^. LAMP recognizes six to eight regions of the DNA and utilizes four to six primers, a strand-displacing DNA polymerase, and an additional reverse transcriptase in case of RNA amplification (i.e., RT-LAMP). The result is a highly specific, exponential amplification of the target nucleic acid in 20–60 min^22^. This extensive synthesis facilitates the detection of the amplicon via a variety of techniques, which include the agarose-gel^23^, real-time fluorescence detection using an intercalating DNA dye^24^, turbidity^25^, metal-sensitive indicator dye^26^, or a pH-sensitive indicator dye in minimally-buffered or non-buffered solutions^27,28^. Moreover, there is no need for specialized detection equipment for the latter since direct visual evaluation is possible. Among these detection techniques, the pH-sensitive dyes are the most favorable and convenient in allowing the LAMP to be used in a POC setting. Their underlying principle is that a successful amplification produces hydrogen ions as a byproduct, which changes the initially alkaline solution to an acidic solution and reduces the pH value (by ≥ 2 pH units). This drop in the pH value is detected by a change in the color of a pH sensitive dye that is added with other reagents^27,29^. A detailed comparison of the molecular and non-molecular techniques for diagnosing COVID-19 is provided in Table 1.

**Table 1:** The comparison of selective molecular and non-molecular techniques for the detection of COVID-19.

Sr		Molecular Test		Anti-body Test		Antigen Test
		RT-PCR	RT-LAMP	ELISA	IgG/IgM Lateral Flow Assay	
1	What it detects:	Viral RNA	Viral RNA	Antibody	Antibody	Viral Antigens (Specific proteins on the surface of virus)
2	Sample taken from:	Nasopharyngeal Swab, sputum, saliva, stool	Same as RT-PCR	Blood	Human serum, plasma, or whole blood	Nasal or throat swab
3	Performed in:	Lab	Lab or Point-of-care	Lab	Point-of-care	Lab
4	Time Required:	3–4 h	Variable (35 min–3 h)	1–3 h	10–20 min	15 min
5	Specificity:	High	High	High (after at least 14 days of active infection)	High (after at least 14 days of active infection)	Moderate
6	Sensitivity:	High	High	High (after at least 14 days of active infection)	High (after at least 14 days of active infection)	Moderate
7	What it tells	Active coronavirus infection	Active coronavirus infection	Past coronavirus infection	Past coronavirus infection	Active coronavirus infection
8	Pros:	Commonly used; gold standard	Rapid Results can be detected by naked eye	Simple and cheap	Simple; cheap; fast; visual inspection possible	Positive results are usually highly accurate
9	Cons:	Requires bulky, expensive, and specialized equipment to analyze the results The time needed to complete the test is high; trained personnel is required	The design of primers can be complex; more chances of primer-to-primer interaction Qualitative test (challenging to quantify the results, i.e., the level of viral infection)	Not well established; it can take from days to several weeks to develop antibodies enough to be detected	Does not show active coronavirus infection Needs a PCR validation	A higher chance of missing an active infection (less sensitive than molecular tests); negative results may need to be confirmed via a molecular test
10	Cost	High	Moderate	Low	Low	Low

Due to these positive attributes and potential benefits, there is a growing interest in using the LAMP technique in several fields and POC diagnostics of various pathogens, especially the SARS-CoV-2^20,22,27,30–32^. A CMOS-based RT-LAMP POC platform was reported to amplify/detect the nucleocapsid (N) gene of SARS-CoV-2^33^. The platform is integrated with a smartphone for data visualization and presents the results within 20 min after RNA extraction. An additively manufactured portable POC platform detected the presence of SARS-CoV-2 in 30 min without requiring the RNA extraction step^20^. The samples were first thermally lysed, followed by mixing samples with RT-LAMP reagents in the serpentine microfluidic cartridge. During the amplification step, the mixture is heated at 65 °C, and fluorescence emission during the process is recorded using a smartphone camera integrated with the cartridge. In addition, a tablet PC-based POC colorimetric platform to detect COVID-19 was also introduced^34^. The device could perform 8 tests simultaneously and yield qualitative results in ~ 30 min. In November 2020, the US Food and Drug Administration (FDA) has issued an Emergency Use Authorization (EUA) to an RT-LAMP-based POC device ‘Lucira’^35^ for the qualitative detection of COVID-19. The single-use device can be used outside a lab setting by individuals of 14-year-old or older, using self-collected nasopharyngeal swab samples. Owing to the ease in operation and their affordability, more devices of similar nature are expected to be introduced in the near future.

The subject of utilizing deep learning for early detection and prediction of COVID-19 has also attracted considerable attention since the pandemic outbreak. Researchers have developed numerous classification algorithms across the globe to enable fast and reliable detection of the infection^36^. Efforts are on the way to using deep-learning-based detection/prediction in conjunction with the existing diagnostic tools to produce more accurate and time-efficient results. This is expected to assist clinicians and healthcare professionals in making more appropriate and timely data-driven decisions. Furthermore, automated diagnostics is more likely to eliminate human-related errors in the analyses, thus facilitating both patients and the health care systems.

Most of the deep-learning studies investigated the chest information within the healthy and COVID-19 positive subjects^37^. These chest manifestations are obtained either through Computed Tomography (C.T.)^38–40^, X-rays^41,42^, or fused C.T. and X-ray imagery^43,44^. The work of Wang et al.^40^ is notable here as they used DenseNet-201^45^ driven encoder-decoder network to extract chest lesions from the C.T. imagery. Moreover, the extracted lesions are then utilized in giving the lesion-aware COVID-19 diagnostic and prognostic analysis. The authors tested their framework on a total of 5,372 C.T. imagery, where it achieved the area-under-the-curve (AUC) score of 0.86 and 0.88 for identifying COVID-19 from viral and other pneumonia, respectively. Similarly, Xu. et al.^41^ developed a custom multi-class deep classification model that takes patches of the candidate C.T. scan to screen the presence of COVID-19, and other chest abnormalities, such as Influenza-A-Viral Pneumonia (IVAP) and Irrelevant to Infection (ITI) groups. The framework achieved an accuracy rate of 0.8670 when tested on a dataset containing 11,871 C.T. image patches (from which 2634 patches show COVID-19 symptoms, 2661 patches contain IVAP pathologies, and 6576 belonged to the ITI group). It should also be noted that the processing time for the C.T. scans is quite less; however, it requires expensive and complicated equipment. On the other hand, the X-ray imagery (particularly CXRs) costs less and has lesser memory requirements. Considering this aspect, Chowdhury et al.^42^ tuned the pre-trained models (such as ResNet-101^46^, DenseNet-201^45^, MobileNetv2^47^, etc.) to screen healthy, COVID-19 pneumonia, and viral pneumonia from the 3487 CXRs and achieved the best accuracy of 0.9970 through DenseNet-201^45^. Similarly, Narin et al.^44^ utilized pre-trained models to screen healthy and infected (with COVID-19 pneumonia, bacterial pneumonia, and viral pneumonia) patients. Their model achieved an overall accuracy of 0.9610, 0.9950, and 0.9970 on the three custom datasets consisting of 3141, 1843, and 3113 CXRs, respectively. Islam et al.^48^ utilized CNN coupled LSTM model to detect COVID-19 manifestation from the CXR imagery. They validated their framework on a custom dataset containing 4575 scans and achieved the AUC and accuracy ratings of 0.9990 and 0.9940, respectively. Saha et al.^49^ proposed EMCNet, a deep feature extractor-based ensemble of different classification models, to diagnose COVID-19 via CXR imagery. EMCNet was tested on 460 CXRs where it achieved its accuracy, sensitivity, and precision ratings of 0.9891, 0.9782, and 1.0000, respectively. Islam et al.^50^ combined CNN backbones such as ResNets^46^, DenseNets^45^, etc., with recurrent neural networks to effectively recognize healthy, COVID-19 pneumonia and non-COVID-19 pneumonic pathologies from the CXR imagery. They tested their framework on a dataset consisting of 1,388 CXRs where the framework achieved the best accuracy of 0.9986 by coupling VGG-19^51^ with RNN. Moreover, Islam et al.^52^ presented a review of different modalities that are majorly used in conjunction with the deep learning systems towards screening and grading COVID-19 manifestations. Asraf et al.^53^ presented an overview of the application of deep learning schemes to control the COVID-19 spread. Rahman et al.^54^ discussed four different applications of machine learning approaches to combat COVID-19 and its related challenges. Azmat Ullah et al.^55^ presented a review of scalable telehealth services that supports patients suffering from COVID-19. Islam et al.^56^ discussed the wearable monitoring devices (driven via respiration rate, heart rate, temperature, and oxygen saturation levels), and respiratory support systems that are frequently used in assisting COVID-19 positive subjects. In another work, Islam et al.^57^ presented an overview of breathing aid devices such as ventilators, and continuous positive airway pressure that aids in rehabilitating the COVID-19 subjects.

In addition, Batista et al.^58^ utilized 235 RT-PCR samples to screen COVID-19 via different machine learning models, such as Support Vector Machines (SVM), Random Forests (RF), Artificial Neural Networks (ANN), Logistic Regression (LR), and Gradient Boosting Trees (GBT). They achieved the best AUC score of 0.847 using SVM and RF. Jiang et al.^59^ used predictive analysis based on the acute respiratory distress syndrome, alanine aminotransferase, elevated hemoglobin, and myalgias and achieved the accuracy of 0.800 to screen RT-PCR samples of 53 subjects as healthy or COVID-19 positive. Finally, Rahman et al.^60^ developed a custom lightweight CNN model to detect persons with face mask violations in smart city networks through closed-circuit television (CCTV) imagery. They tested their model on a local dataset containing 308 scans where they achieved the accuracy of 0.9870 towards accurately recognizing the persons with and without face masks.

Looking into the literature, we can observe that many researchers have worked on screening COVID-19 via deep learning. The majority of these methods rely on finding clinical manifestations from the CXRs. However, the assessment of COVID-19 from CXR is vulnerable to noise and other vendor artifacts^61^. Furthermore, the clinical biomarkers within CXRs for diagnosing the COVID-19 and non-COVID-19 pneumonia are highly correlated, which can affect the performance of deep learning system^62^. It should be noted that the COVID-19 screening through C.T. imagery or fused C.T. and CXR imagery is reliable^40^. Nevertheless, incorporating C.T. imagery for rapid COVID-19 analysis is costly and cannot be performed in remote clinics and hospitals. To overcome these limitations, we present a cost-effective device that can simultaneously acquire Reverse-Transcription Loop-Mediated Isothermal Amplification (RT-LAMP) reactions and utilize a multi-resolution deep classification model to screen those reactions as healthy and COVID-19 positive accurately. A detailed discussion on the novel contributions of the proposed system is presented in the subsequent section.

### Contributions

In this paper, we report a low-cost, portable, and re-usable deep learning-driven instrument (dubbed KU-LAMP) that performs RT-LAMP reactions for the rapid detection of COVID-19 pathologies. The device is fabricated using 3-D printing technology and is powered by a single 5-V AC-DC adapter. Capable of performing 16 simultaneous reactions, KU-LAMP displays the presence of SARS-CoV-2 RNA in the positive samples, which the proposed multi-resolution deep classification model automatically detects. Moreover, the device can also sustainably perform multiple sets of tests. In the current work, the capability of KU-LAMP in detecting COVID-19 was demonstrated for 319 locally acquired samples. Furthermore, the current protocol obviates the need for the time-consuming RNA extraction step which is performed separately with the majority of the commercial kits. Thus, a single platform (KU-LAMP), combining the RNA extraction and RT-LAMP amplification steps, allows for the qualitative detection of COVID-19 from nasopharyngeal samples in only 45 min (10 min for pre-heating and 35 min for RT-LAMP reactions) starting from sample preparation till the analysis of LAMP products. The developed testing system will substantially enhance the testing capacity and thus help in meeting the overwhelming demand for rapid and accurate COVID-19 diagnosis. To summarize, the main contributions of the paper are:

- To the best of our knowledge, this is the first attempt that couples RT-LAMP with deep learning to autonomously detect the presence of COVID-19 pathologies via RT-LAMP reaction samples eliminating the need for a separate RNA extraction step and additional optical modules.
- The KU-LAMP provides an efficient way to screen COVID-19 within 45 min after procuring the nasopharyngeal sample from the candidate subject. This contrasts with the conventional RT-PCR method, which takes, on average, more than three hours while having competitive detection performance.
- The diagnostic capacity of the KU-LAMP has been evaluated in the clinical settings, where it achieved the statistically significant Pearson correlation coefficient of 0.8032 and Cramer’s $\varphi$ coefficient of 0.4122 against the clinician grading. This indicates that the KU-LAMP can be utilized as an assistive point-of-care unit for the autonomous and rapid screening of COVID-19 as per the clinical standards.

## Materials and methods

This section presents a thorough insight into the materials that we used to design the KU-LAMP. Furthermore, this section also presents a detailed overview of the proposed method through which we first acquire the samples of the candidate subjects (in real-time). Afterward, the obtained samples are passed to the proposed classification model, which extracts discriminative feature representations (from the input RT-LAMP scans) to screen COVID-19.

### Materials

#### Device design

**Figure 1:**
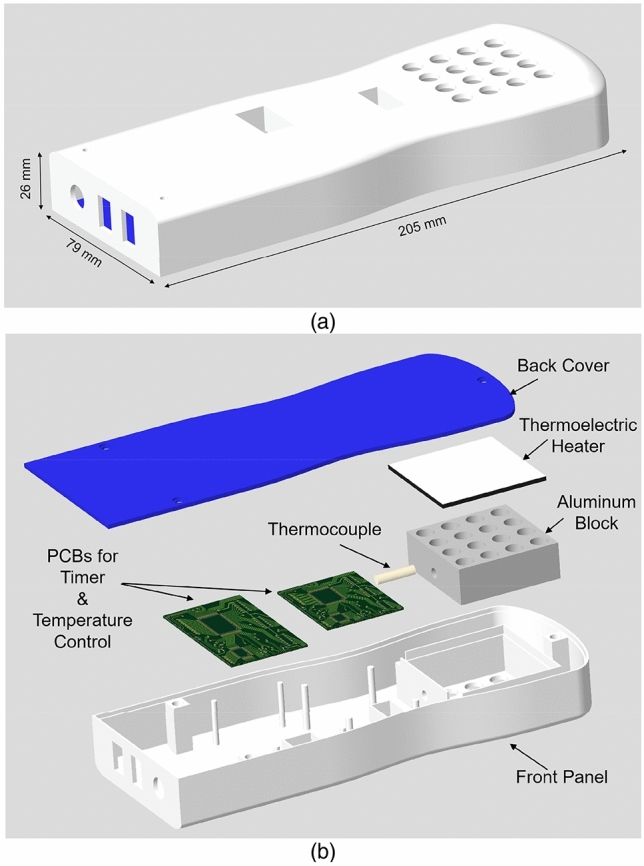
(**a**) Schematic diagram of KU-LAMP showing the dimensions of the device that allows processing of 16 samples simultaneously. The dimensions of the device are 205 mm (l) × 79 mm (w) × 26 mm (h). (**b**) An exploded view of the device showing the components. The figure is generated in PTC Creo CAD software.

Before realizing the device using the 3-D printing technology, it was first designed using Creo Parametric 6.0.3.0. Figure 1 displays the design and parametric views along with the dimensions. The dimensions of the device are 205 mm × 79 mm × 26 mm. As shown in Fig. 1, the device comprises 16 slots in which the 700 µl tubes can be inserted to perform the RNA extraction via direct lysis, followed by the RT-LAMP amplification. A single reaction temperature of 65 °C is required to achieve the lysis, reverse transcription step, and nucleic acid amplification. This temperature-dependent activation calls for a uniform temperature distribution throughout the sample compartment, which is ensured by an efficient thermal design. In an effort to fulfill this requirement, the sample compartment is made of an aluminum (Al) block with holes, wherein a thermally conductive liner (DOWSIL 3-6655), in the form of a sample tube, is cast inside each hole. The Al block, together with the thermally conductive liner, serves as an efficient heat transfer medium between the solid-state thermoelectric heater (Laird Thermal Systems, Inc) located at the bottom of the block and the tubes containing the samples. The remaining space in the KU-LAMP device is utilized to house the other components required to perform the amplification, including a heat controller, DC-DC regulator, switches, and a timer with a buzzer to indicate the completion of the amplification process.

#### Thermal modeling of the device

**Figure 2:**
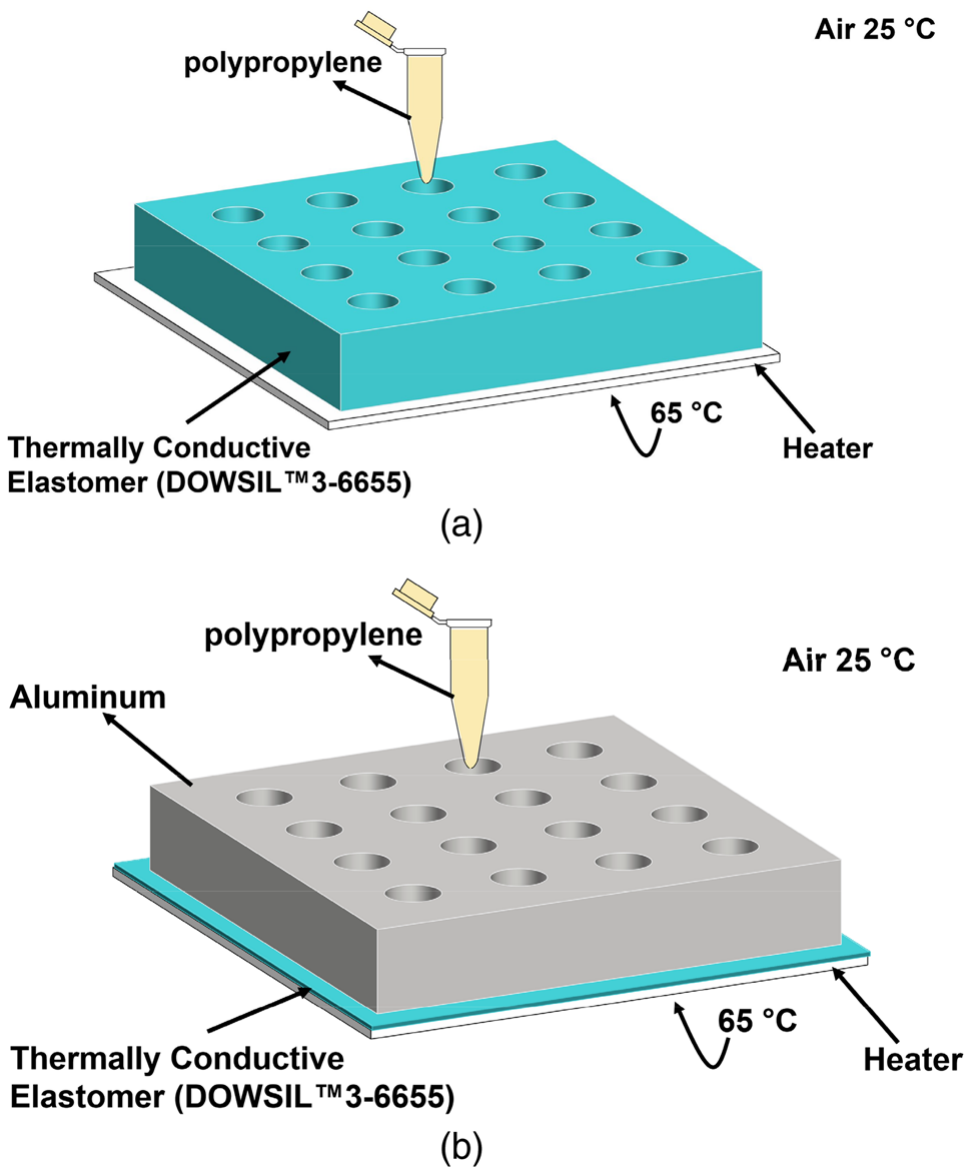
(**a**) Schematics of the designs used in the thermal modeling for KU-LAMP with the details of material and boundary conditions. The sample compartment block is made from (**a**) thermally conductive material (i.e., DOWSIL 3-6655 and (**b**) aluminum with a thin layer of DOWSIL 3-6655 underneath and inside the holes, as a liner. A sample tube is shown for illustrative purposes. The figure is generated in COMSOL Multiphysics software.

Indeed, the temperature is one of the critical determinants of specificity in nucleic acid amplification techniques, as high temperatures could cause false-positive results through nonspecific amplification^63^. To ensure that the temperature is maintained at around 65 °C within each sample tube, the temperature distribution within the sample compartment was simulated in 3 D using the software COMSOL Multiphysics. A time-dependent study using the ‘Heat Transfer Module’ in COMSOL Multiphysics was performed in each case to simulate (i) the heat conduction within the compartment and (ii) the natural convection of heat from the outside surfaces of the compartment to open air. Two different designs for the sample compartment were investigated to compare their heat transfer characteristics as a function of time. In the first design, the sample tubes were placed in a block made of the thermally conductive material (i.e., DOWSIL 3-6655), whereas the latter design used an Al block and a thin layer of DOWSIL 3-6655 placed between the Al block and heater. Moreover, DOWSIL 3-6655 was utilized to fill the gap between the conical form of the sample tubes and holes opened in the block. Figure 2 shows the schematics of both designs. The bottom surface temperature was set to 65 °C, which is the pre-set temperature output of the solid-state thermoelectric heater controlled by a temperature controller (Laird Thermal Systems, Inc). In addition, continuity boundary condition was used between the blocks and the surrounding air. This setup allows the top and sidewalls of the blocks to be at a temperature consistent with the actual temperatures measured at the exact locations using a thermometer (Fluke, 51 Series II). Further, a ‘Heat Flux’ boundary condition was applied to the top and sidewalls of the compartment and the tubes. This boundary condition utilized an in-built convective heat transfer coefficient correlation function and ambient temperature (i.e., 25 °C) to simulate the heat flux from the walls to ambient air.

**Figure 3:**
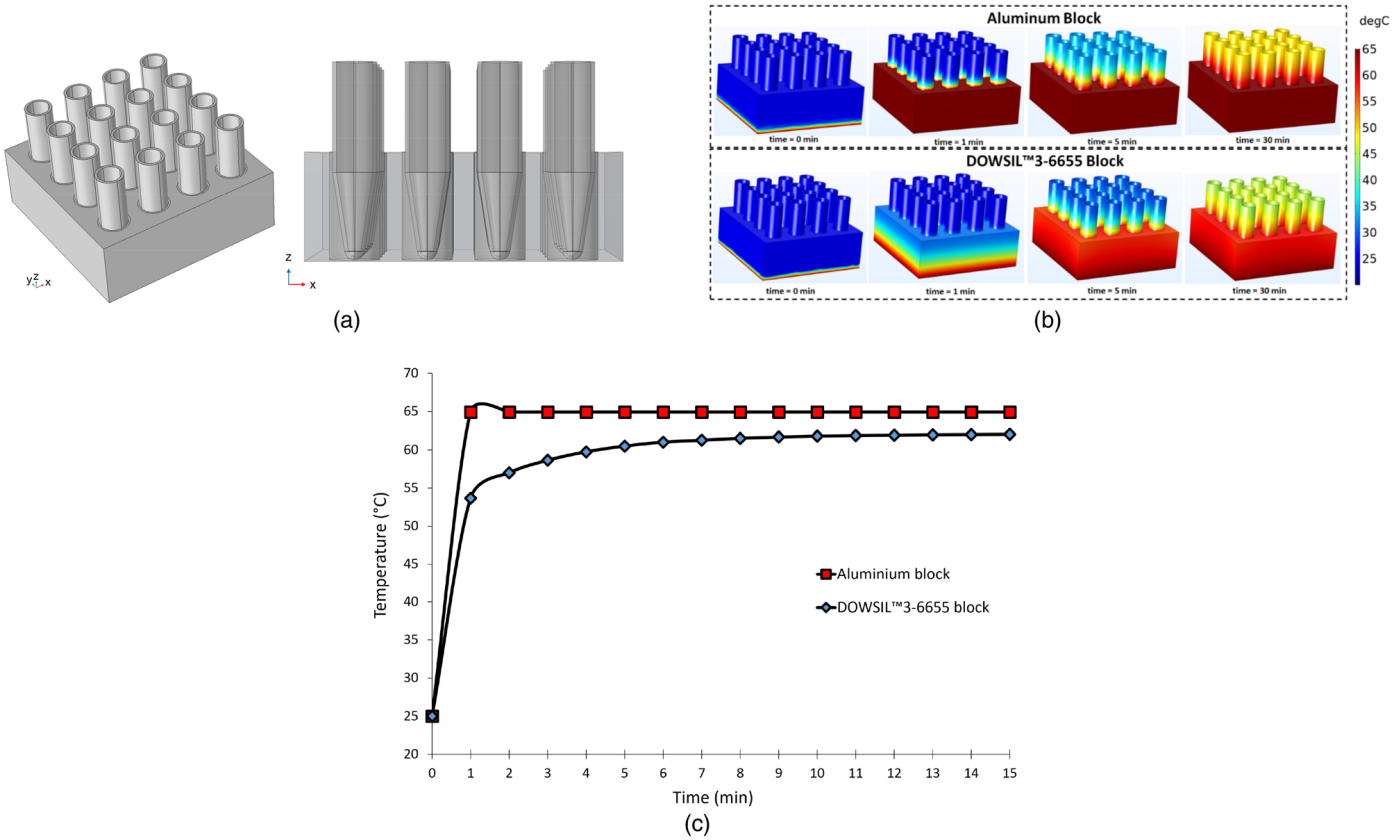
(**a**) The schematics of the model setup in COMSOL Multiphysics with 16 tubes inserted in the block. (**b**) Temperature distribution in the complete system for t = 0, 1, 5, and 30 min. (**c**) Temperature profile monitored over 15 min at a point on the upper surface of the block. The figure is generated in COMSOL Multiphysics software.


This simple model provides a powerful and accurate method to compute the temperature distribution and heat transfer rate without including a large volume around the volume of interest to represent the air domain. Thus, the computational load of the simulation is reduced significantly^64^. The thermal properties of Al provided in the COMSOL material library were used, in addition to the user-defined properties for the thermally conductive material of the liner (heat capacity at constant pressure Cp = 920 J/kg K; density ρ = 2700 kg/m^3^; thermal conductivity k = 1.8 W/m K). The sample tubes (Eppendorf, 0.7 ml) used for the incubation are made of polypropylene (Cp = 1800 J/kg.K; ρ = 920 kg/m^3^; k = 0.11–0.44 W/m K)^65^. Figure 3 displays the model setup in COMSOL Multiphysics (Fig. 3a) and the results of the time-dependent simulations (Fig. 3b,c). The results reveal the temperature distribution in the entire sample compartment, including the tubes (Fig. 3b), as well as the temperature profiles at a point on the upper face of the compartment (Fig. 3c), for both cases and for 30 min. It can be seen that the design employing Al along with a thin layer of DOWSIL 3-6655 attains the desired temperature (65 °C) within a minute. On the contrary, the purely DOWSIL 3-6655 block could not be completely heated up to 65 °C even after 30 min. Due to its superior thermal performance, the design shown in Fig. 2b was employed in KU-LAMP. The chosen design had the added advantage of reducing the overall weight of the device as well. It should be noted that the temperature near the cap of the tubes is still less than 65 °C; however, it does not affect the performance of the system, since the reaction mixture covers only a minute fraction of the tube volume and is completely immersed in the Al block, where the temperature remains uniform at 65 °C.

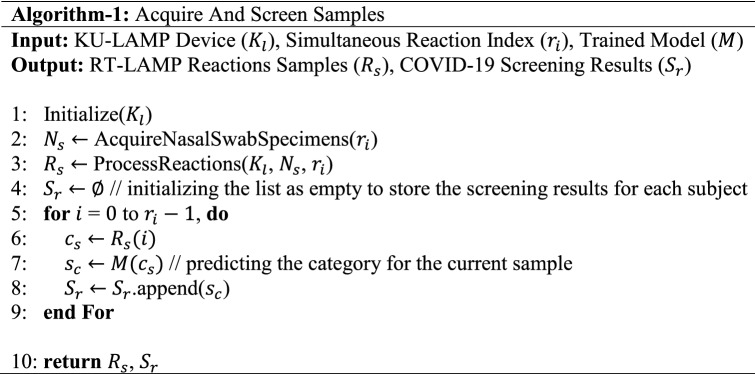


### Methods

**Figure 4:**
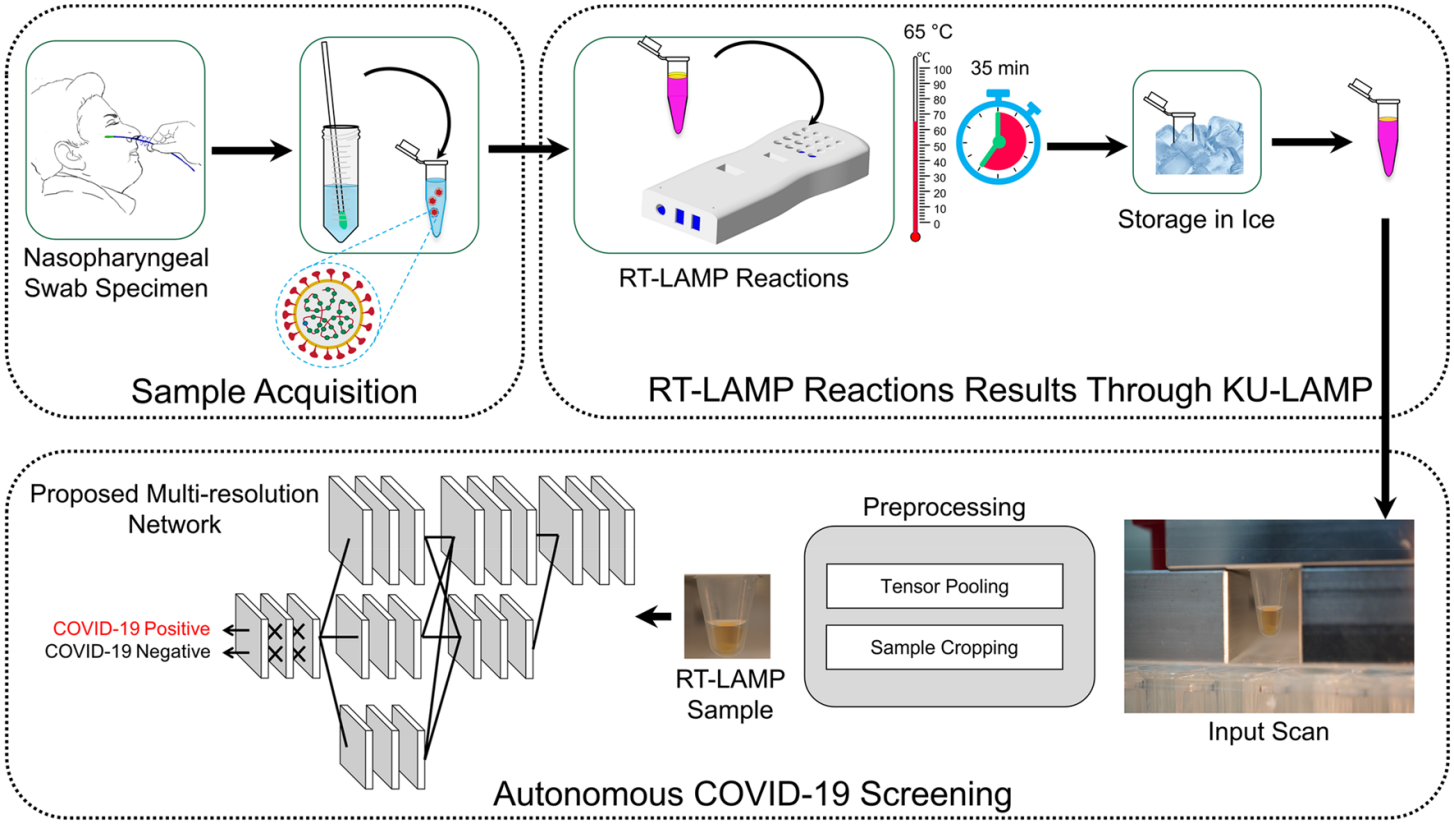
The block diagram of the proposed system pipeline. The figure is created in MS powerpoint.

The overall block diagram of the proposed system is shown in Fig. 4, whereas the complete working schematic of the proposed framework is depicted in Algorithm-1. Here, it can be observed that, at first, the nasopharyngeal sample of the candidate subjects is obtained. Afterward, the acquired sample is passed to the KU-LAMP where it is pre-heated to perform the RNA extraction. After extracting the RNAs, the RT-LAMP reaction is initiated, and the result of the reaction is captured through the mounted camera. The scan (containing the reaction results) is passed to the proposed multi-resolution classification network for screening the presence of COVID-19. The detailed discussion on each of the modules (within the proposed system) is discussed below:

#### Sample acquisition

As mentioned earlier, the complete process following the collection of nasopharyngeal samples till the detection of SARS-CoV-2 RNA in KU-LAMP is performed in 45 min only. The short processing time of our device is a direct consequence of the elimination of the RNA extraction step, which is replaced by 10 min heat lysis of samples performed on KU-LAMP at 65 °C, the same temperature as the LAMP reaction. Most commercial kits require extracted pure RNA as the input for the amplification step. Thus, additional extraction steps are performed in the conventional process, including lysis, RNA isolation, and removal of the inhibitory agents. The more popular and traditional RNA extraction methods involve using magnetic beads and/or chemical solutions to isolate the RNA from the samples. Hence, these methods increase the processing time and add complexity to the process.

#### RT-LAMP reaction results through KU-LAMP

**Figure 5:**
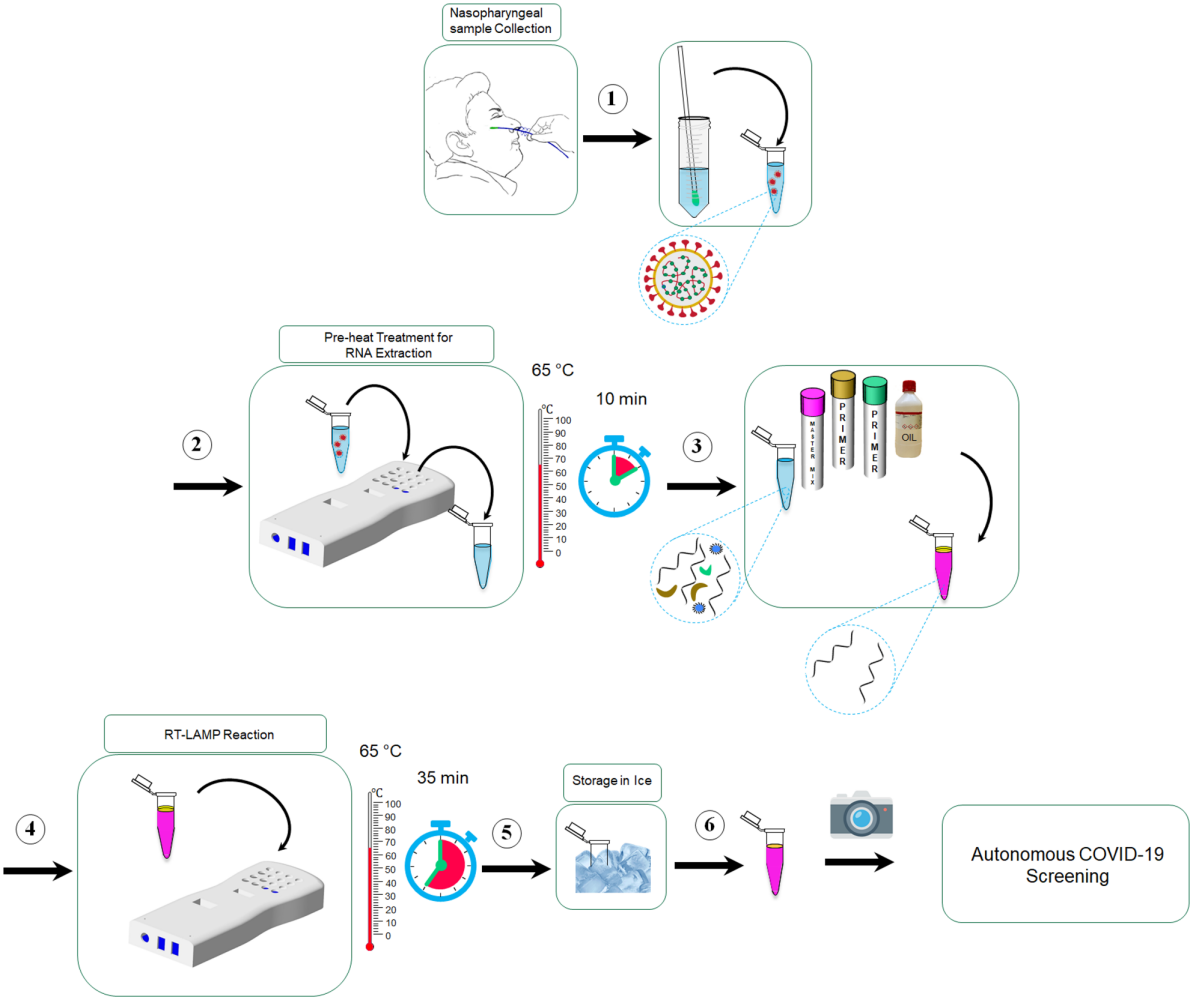
Schematics of the steps comprising the protocol for COVID-19 detection using KU-LAMP. (The figure is generated in MS powerpoint).

The complete schematic diagram of the experimental protocol followed in the current work is shown in Fig. 5. It should be noted that the methods and acceptance criteria to demonstrate that an assay is valid and appropriate for its intended application have been established by the College of American Pathology (CAP) and Clinical Laboratory Improvement Amendments (CLIA). All experiments in the current work were performed in accordance with relevant guidelines and regulations. The experiments fulfill local (Health Authority Abu Dhabi-HAAD) and international (College of American Pathologists-CAP) requirements for operation and accreditation purposes. Furthermore, all the COVID-19 samples were obtained from major hospitals and quarantine areas in Abu Dhabi, UAE. An informed written consent form was obtained from all the participants and their legal representatives of minors in accordance with the Declaration of Helsinki. The complete experimental procedure along with the sample collection was approved by the local ethics committees at Abu Dhabi, namely Health COVID-19 Research Ethics Committee and SEHA Research Ethics Committee.

As the first step of the developed protocol, ~ 30 µl of the nasopharyngeal (swab) sample is placed in a 700 µL Eppendorf tube, and the tube is pre-heated at 65 °C for 10 min using KU-LAMP. This pre-heating step lyses the cells and viral capsid to release the organelles including the viral RNA. The current work aims to detect the target regions from two genes—nucleocapsid (N) and envelope (E) genes—from the SARS-CoV-2 viral genome. In the next step, 2.0 µL of the lysed sample is added to the mixture containing 12.5 µL of WarmStart Colorimetric LAMP 2X Master Mix (New England BioLabs), 5.5 µL of nuclease-free Water, 2.5 µL of the target-specific primer mix for N and E genes, and 2.5 µL of Guanidine Hydrochloride. The presence of guanidine hydrochloride has been proven to significantly increase the speed of the reaction, thus leading to shorter reaction times^66^. The mixture which is prepared in a 700 µl Eppendorf tube is then vortexed to mix all the reagents. Finally, 5 µL of silicone oil is added to the mixture, which encapsulates the mixture at the top and prevents evaporation. The insulating top silicone oil layer also serves to enhance the circulation of the mixture underneath. Consequently, better mixing is obtained during the amplification process. The tubes containing the prepared mixture are then placed in the designated slots of KU-LAMP pre-heated at 65 °C via the solid-state thermoelectric heater and its controller, as described earlier. The incubation at 65 °C continues for 35 min, monitored by a timer and an alarm incorporated into the device. At the end of 35 min, the tubes are removed from the device and immediately placed on ice for about 30 s to stop the reaction.

#### Autonomous COVID-19 screening

**Figure 6:**
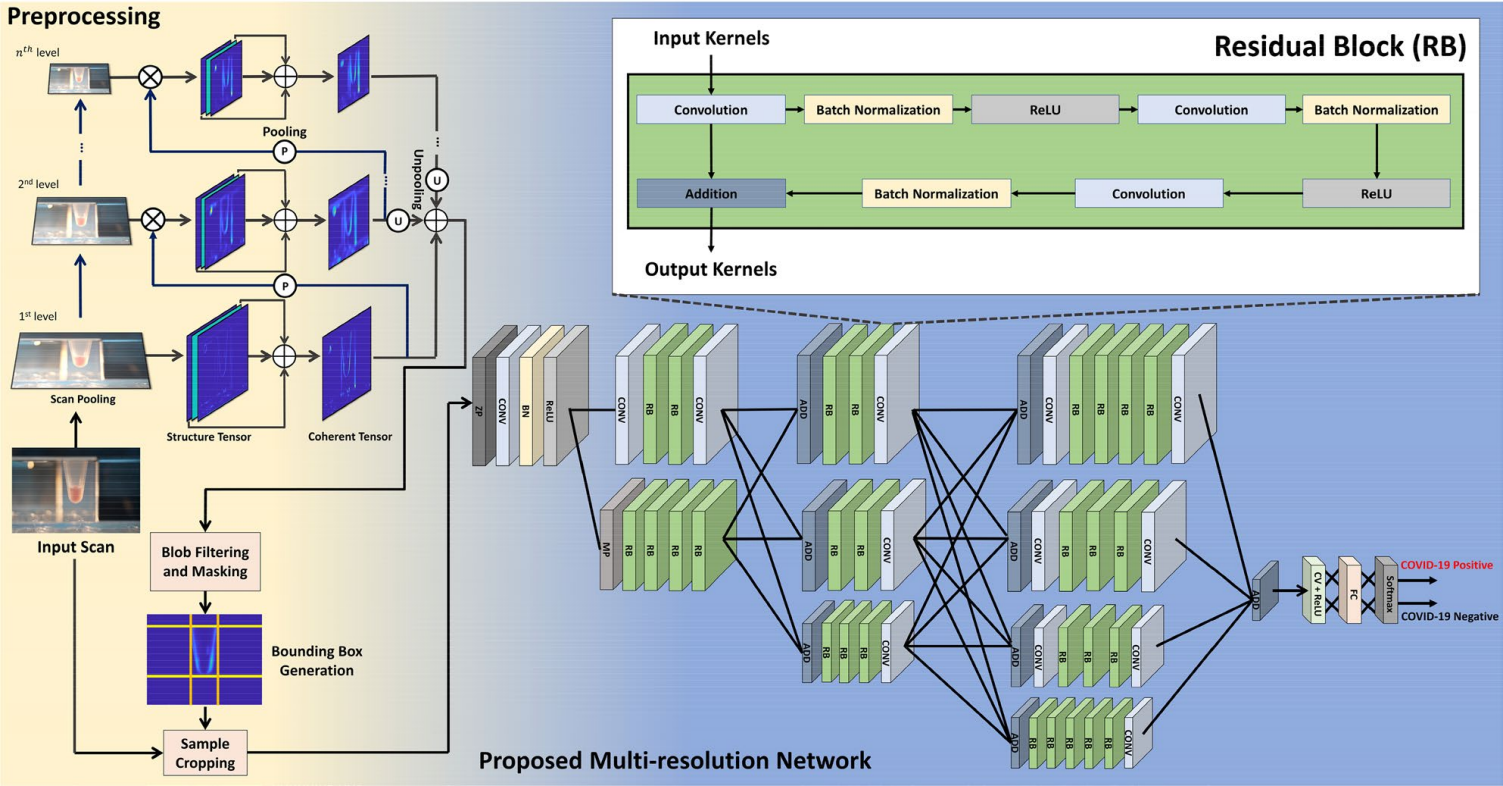
The proposed COVID-19 screening system. When the input scan is passed to the screening block, it is first pre-processed to remove the background artifacts. Afterward, the preprocessed scan (contain RT-LAMP sample) is passed to the proposed multi-resolution network that extracts discriminative features to differentiate COVID-19 vs. non-COVID-19 subjects. Moreover, the abbreviations within the proposed network are: ZP: Zero-padding, CONV: Convolution, BN: Batch Normalization, ReLU: Rectified Linear Unit, RB: Residual Block, MP: Max pooling, ADD: Addition, CV + ReLU: Convolution layer with ReLU activation, and FC: Fully Connected. (The figure is generated in MS Powerpoint).

After the completion of RT-LAMP amplification reactions, we acquired their images which are then passed to the pre-processing stage. The pre-processing step enables us to crop the region-of-interest (containing the reaction sample) from the whole scan using the tensor pooling module^67^. Moreover, after preprocessing the candidate scan, it is passed to the custom multi-resolution network which extracts discriminative features from it to screen the candidate subject against COVID-19. The detailed description of the pre-processing stage and the proposed multi-resolution model is shown in Fig. 6 and discussed below.

##### Preprocessing

Apart from the RT-LAMP output sample, the raw input scan also contains artifacts and background information, leading to false positives while screening the COVID-19 pathologies. To overcome these misclassifications, we integrate a pre-processing block (within the proposed system) that suppresses the background information while highlighting the maximum transitions within the candidate scan (and these maximum transitions belong to the RT-LAMP samples since the intensity of their pixels produces maximum differences with the background pixels). Therefore, the pre-processing (within the proposed framework) is performed through a tensor-pooling module^67^ that analyzes the strength of the transitional information (related to the RT-LAMP samples) while suppressing the background information.

##### Tensor pooling module

The tensor pooling module shrinks the candidate image into *n* pyramids. At each pyramid level, the transitional information of the RT-LAMP sample is analyzed with respect to the background regions, and it is highlighted (within the tensors) accordingly. Moreover, at each pyramid decomposition step, the lower spectral components of the image content are retained, whereas the higher frequency content are clipped. Therefore, the higher-order to the pyramid decomposition level only retains the lower frequency content, which, when added together with the output of each pyramid block, highlights the contours of the higher transitional content, as shown in Fig. 4. Also, at each pyramid level, the transitional information of the candidate scan is analyzed through multi-oriented structure tensors^68,69^ that compute *N* × *N* symmetric block-structured matrix through *N* gradients (of the candidate scan) across N orientations, as expressed below:

1 $$\left[\begin{array}{ccc}\varphi *({\nabla }^{0}{\nabla }^{0})& \cdots & \varphi *({\nabla }^{N-1}{\nabla }^{0})\\ \vdots & \ddots & \vdots \\ \varphi *({\nabla }^{0}{\nabla }^{N-1})& \cdots & \varphi *({\nabla }^{N-1}{\nabla }^{N-1})\end{array}\right],$$

Each tensor ($\varphi *({\nabla }^{i}{\nabla }^{j})$) in Eq. 1 is generated by computing the outer product of image gradients with the smoothing filter $\varphi$, where the orientation ($\vartheta$) of the gradients ${\nabla }^{k}$, is calculated using $\frac{2\pi k}{N}$ in which $k$ goes from 0 to $N$-1 s. Furthermore, it should be noted that only $\frac{N(N-1)}{2}$ Comp tensors within the matrix (in Eq. 1) are unique because the matrix is symmetric. From these unique tensors, we obtain the coherent tensor, reflecting the RT-LAMP reaction sample’s predominant orientations, by adding the most insightful tensors (out of $\frac{N(N-1)}{2}$ tensors), which are selected based upon their norm.

Moreover, after obtaining the final tensor representation of the candidate scan, we perform blob filtering and masking in order to remove noisy outliers. Afterward, we generate the bounding box around the RT-LAMP output sample by analyzing the minimum and maximum transition of the sample in both image directions. Through this bounding box, we crop the original image and feed it to the proposed multi-resolution network for COVID-19 screening.

##### Proposed multi-resolution network

After acquiring the pre-processed scan, it is passed to the proposed multi-resolution model for COVID-19 screening purposes. We want to emphasize that the main contribution of this paper is not in the deep learning territory. Instead, it is in the rapid and portable acquisition of COVID-19 samples through the Reverse-Transcription Loop-Mediated Isothermal Amplification (RT-LAMP) scheme. Moreover, the purpose of coupling deep learning with the proposed device (KU-LAMP) is to provide an end-to-end detection of COVID-19 from the acquired RT-LAMP samples. Having said that, the deep learning architecture which we employ in the proposed system is also an improved version of the high-resolution networks (HRNets)^70^, i.e., the original HRNets employs 1 × 1 convolutions to generate latent feature representations from the high-resolution channels. The 1 × 1 convolutions are costly in computational resources^70^ and thus cannot be utilized on embedded devices. Furthermore, feature space representations obtained from the high-resolution channels (like in HRNets^70^) are susceptible to noise and vendor artifacts^71^. Therefore, they cannot be employed directly within the critical healthcare system (primarily related to COVID-19 screening). To overcome these limitations, we proposed a multi-resolution COVID-19 screening network that, instead of decomposing the feature maps sequentially, extends parallel pipelines that generate distant feature representations of the RT-LAMP sample across various scales in order to effectively preserve the contextual information about the RNA reactions. Moreover, instead of taking high-resolution features only (like HRNets^70^), the proposed model combines multi-scale features across each depth of the network to effectively generate discriminative latent feature representations of the negative and positive COVID-19 cases to facilitate the classification unit towards accurately screening the underlying sample. Furthermore, unlike HRNet^70^ (which extensively uses 1 × 1 convolutions), the proposed model is lightweight due to the employment of the residual blocks (as shown in Fig. 6), which extends it for real-time deployment on embedded devices like KU-LAMP. Architecturally, the proposed model contains one input layer, 98 convolution layers (both linear and atrous), 85 batch normalization layers, 58 ReLU activations, one max pooling, one zero-padding, one fully connected, one softmax activation, and 36 addition layers with a total of 5.2 M parameters as shown in Table 2. Moreover, the training details of the proposed model are extensively discussed in the Experimental Setup section.

**Table 2:** The number of layers and learnable parameters within the proposed multi-resolution network.

Layers	Number of Parameters
Input	0
Convolution	5,094,760
Batch Normalization	106,240
ReLU	0
Max Pooling	0
Zero-Padding	0
Fully Connected	4098
Softmax	0
Addition	0
Total parameters	5,205,098

## Experimental setup

This section contains detailed experimental details (including dataset information, training protocols, and evaluation metrics), which we followed in this research.

### Dataset details

**Figure 7:**
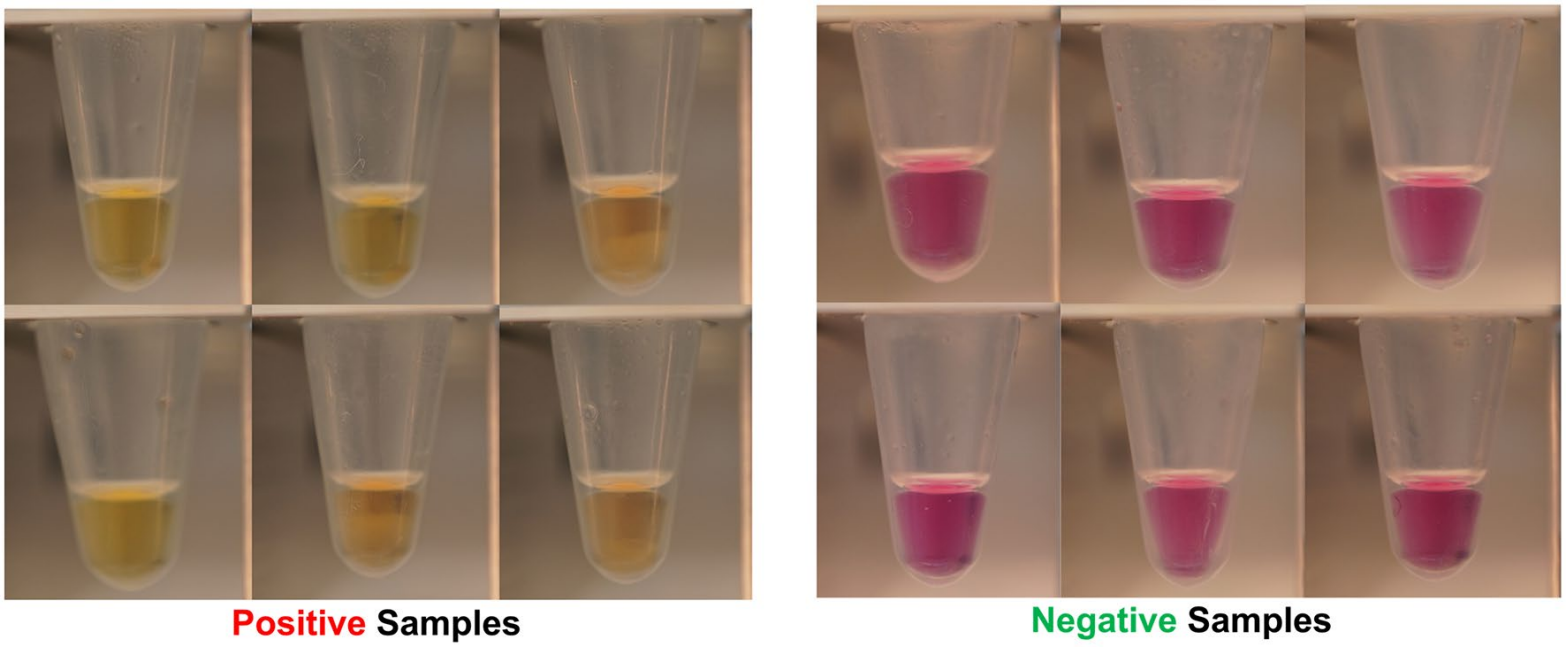
Examples of Positive and Negative COVID-19 Scans within the Proposed Dataset.

**Figure 8:**
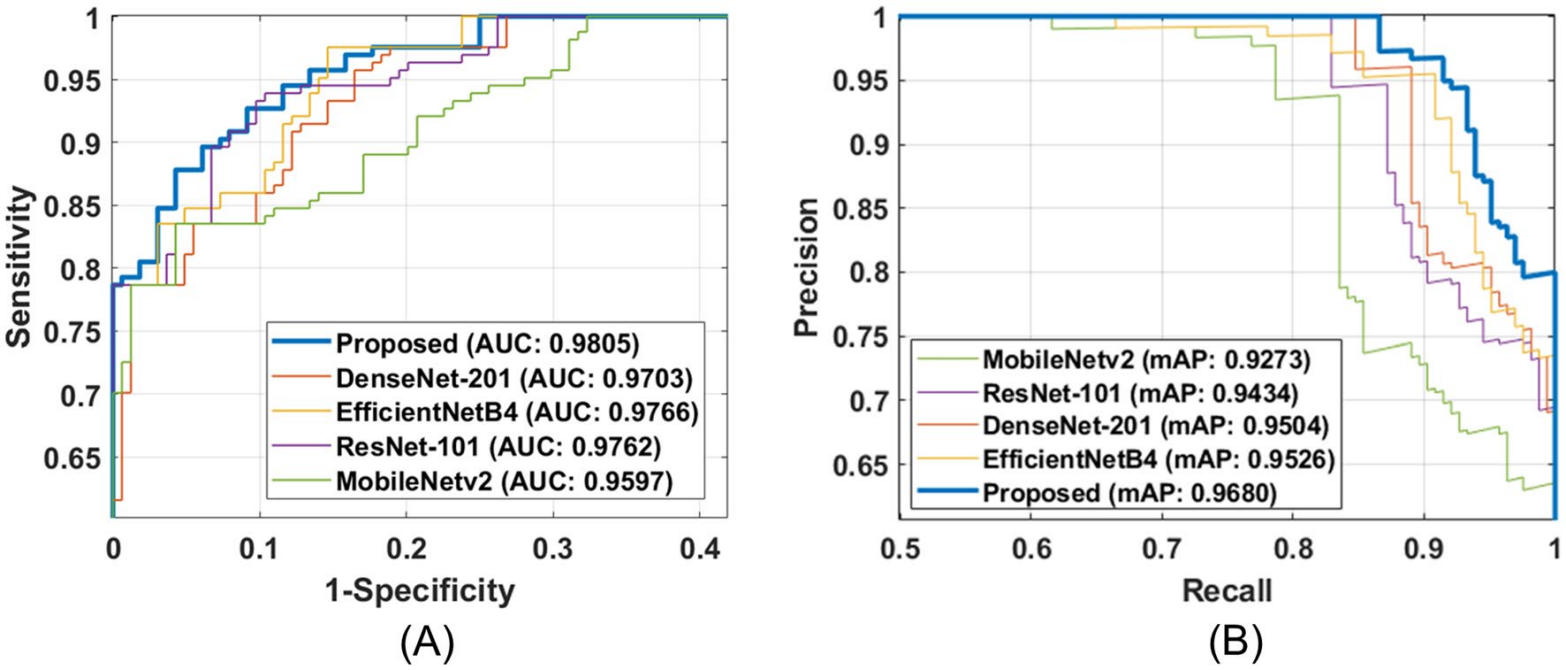
Performance comparison of proposed network with state-of-the-art classification models in terms of (**A**) ROC curve, (**B**) PR curve. (The figure is generated using MATLAB).

To perform autonomous COVID-19 screening, a total of 319 RT-qPCR-validated clinical samples of COVID-19 positive and COVID-19 negative (healthy) subjects were obtained from major hospitals and quarantine areas in Abu Dhabi, U.A.E. Out of these 319 samples, the images for 250 samples were taken that served our local dataset containing for automated COVID-19 screening using deep learning. Some of the examples for both negative and positive COVID-19 cases within the proposed dataset are shown in Fig. 7. Moreover, we also divided the positive cases into four groups based on the cycle thresholds (C_t_) from the RT-qPCR as demonstrated in Fig. 8. Apart from this, out of these 250 samples, 60% (i.e., 148 samples) were used for training (in which 76 reflect negative cases and 72 reflect positive cases). The remaining 40% (i.e., 102 scans) were used for the testing purposes. We also performed data augmentation on the training set to achieve effective disease classification performance at the inference stage. The augmentation steps include flipping the original scan and rotating the original and flip scan from -5 to 5 degrees in step of 1 degree. These steps resulted in the generation of 11,000 scans which we used to train the proposed network for COVID-19 screening tasks.

### Training and implementation details

The proposed network is implemented through TensorFlow 2.2.0, Python 3.7.4 on a machine having Intel Core i9-10940X@3.30 GHz CPU, 128 GB RAM, and an NVIDIA Quadro RTX 6000 with cuDNN v7.5, and a CUDA Toolkit 10.1.243. The training of the proposed model was conducted for 50 epochs with a batch size of 32, and the optimizer used during the training was ADADELTA having the default learning rate of 1 and a decay rate of 0.95. Apart from this, the network loss (in each iteration) is computed via. categorical loss function (${L}_{ce}$) as expressed below:

2 $${L}_{ce}=\sum_{i=0}^{{b}_{s}-1}\sum_{j=0}^{c-1}{t}_{i,j}log(p{}_{i,j}),$$

where ${b}_{s}$ denotes the batch size, $c$ represents the number of categories, ${t}_{i,j}$ denotes the true label for the *i*th sample belonging to *j*th class and ${p}_{i,j}$ denotes the predicted probability of the *i*th sample for the *j*th class.

### Evaluation metrics

To validate the performance of the proposed system, we used standard classification metrics, such as sensitivity, specificity, precision, and F1 score. Furthermore, to compare the performance of the proposed multi-resolution network with state-of-the-art deep learning models, we also area-under-the-curve (AUC) and mean average precision (mAP) scores computed through the receiver operator characteristics (ROC) and precision-recall (PR) curves, respectively. In addition to this, to validate the COVID-19 screening performance of the proposed network against clinician grading, we used a statistically significant Pearson correlation coefficient ($r$) and Cramer’s $\boldsymbol{\varphi }$ coefficient (${c}_{v}$).

### Declaration

The methods and acceptance criteria to demonstrate that an assay is valid and appropriate for its intended application have been established by the College of American Pathology (CAP) and Clinical Laboratory Improvement Amendments (CLIA). All experiments in the current work were performed in accordance with relevant guidelines and regulations. The experiments fulfill local (Health Authority Abu Dhabi-HAAD) and international (College of American Pathologists-CAP) requirements for operation and accreditation purposes. Furthermore, all the COVID-19 samples were obtained from major hospitals and quarantine areas in Abu Dhabi, UAE. An informed written consent form was obtained from all the participants and their legal representatives of minors in accordance with the Declaration of Helsinki. The sample collection has been approved by the local ethics committees at Abu Dhabi, namely Health COVID-19 Research Ethics Committee and SEHA Research Ethics Committee.

## Results and discussion

This section contains detailed experimental results through which we validated the proposed system. Furthermore, this section also showcases the comparison of the proposed system with state-of-the-art deep learning models to perform COVID-19 screening (through RT-LAMP reaction). In addition to this, we present the detailed validation of the proposed system’s performance against clinical samples grading while to pre-process the candidate scans; we empirically chose the hyper-parameters (*n* and *N*) of the tensor-pooling module to be 3 and 4, respectively (which are also recommended in^50^).

### Comparison of proposed network with State-of-the-Art models

In the first series of experiments, we compare the COVID-19 screening performance of the proposed framework with state-of-the-art models such as ResNet-101^72^, DenseNet-201^45^, EfficientNetB4^46^, and MobileNetv2^47^. Here, the purpose of comparing the performance of the proposed system with these models is to see how well the proposed system performs compared to these pre-trained models as they have been extensively used in the literature for screening COVID-19 pathologies^39–44^. Here, we first tuned all of these models using ${L}_{ce}$ loss function on the same training samples. Afterward, we apply all the models on the test dataset to measure their performance. The comparison is reported in Table 3 where we can see that the proposed network outperforms all the other models by 1.45% in terms of sensitivity, 3.56% in terms of specificity, 0.020% in terms of precision, and 0.740% in terms of F1 score. Nevertheless, we can also observe here that the COVID-19 screening performance of all the models are similar. This indicates that the RT-LAMP output sample serves as a good modality to effectively screen COVID-19 via*.* deep learning (other than CXRs and CT scans which are widely used by the deep learning community^61,73^.

**Table 3:** Performance comparison of the proposed network with state-of-the-art classification models. Bold indicates the best performance while the second-best performance (across each metric) is underlined.

Model	Input size	Sensitivity	Specificity	Precision	F1
ResNet-101^72^	224 × 224 × 3	0.9583	0.9333	0.9718	0.9650
DenseNet-201^45^	224 × 224 × 3	0.9444	0.9000	0.9577	0.9510
EfficientNetB4^46^	224 × 224 × 3	0.9583	**0.9666**	0.9857	0.9718
MobileNetv2^47^	224 × 224 × 3	0.9306	0.9000	0.9571	0.9437
Proposed	224 × 224 × 3	**0.9722**	**0.9666**	**0.9859**	**0.9790**

In addition to this, we also compared the performance of the proposed framework with other models in terms of ROC curves. From Fig. 8A and B, we can observe that the performance of all the models is competitive. Nevertheless, the proposed network achieves the overall best AUC and mAP scores of 0.9805 and 0.9680, respectively.

Apart from this, Table 4 indicates the computational parameters required by each network to screen COVID-19. Here, we can see that the proposed network comes in the second place where it requires a modest amount of more parameters than the lightweight MobileNetv2^47^. However, observing the trade-off between classification performance between proposed network and MobileNetv2^47^ (in Table 3), and the computational parameters (in Table 3), we prefer the use of proposed model as it gives 3.74% performance boost at the minimal expense of the computational cost, which can be handled by modern embedded devices.

**Table 4:** Number of parameters in each model. Bold indicates the lightweight model whereas the heaviest model is underlined.

Model	Input Size	Number of Parameters
ResNet-101^72^	224 × 224 × 3	8.4 M
DenseNet-201^45^	224 × 224 × 3	7.9 M
EfficientNetB4^46^	224 × 224 × 3	7.1 M
MobileNetv2^47^	224 × 224 × 3	**4.3 M**
Proposed	224 × 224 × 3	5.2 M

### Proposed network in clinical trials

In this series of experiments, we evaluated the performance of the proposed network in clinical settings where both the proposed network and clinician graded the RT-LAMP reaction output samples acquired through the KU-LAMP. The results are reported in Table 5 in which we can see that for 33 subjects (both healthy and COVID-19 positive), the proposed framework achieved similar results as compared to the clinician, except for case-20, in which the RT-LAMP samples were at borderline (having pinkish color appearance), which made the proposed framework to produce a false negative. Apart from this, we also computed the Pearson correlation ($r$) and Cramer’s $\boldsymbol{\varphi }$ coefficient (${c}_{v}$) between the proposed network and the clinician grading (for the same cases as reported in Table 5). The objective of computing the $r$ and ${c}_{v}$ is to analyze the statistical significance of the proposed network’s diagnostics as compared to the expert clinicians within a clinical setting. From Table 6, we can see that the proposed network achieved *r* = 0.8032 and ${c}_{v}$=0.4122, which is also statistically significant with p < 0.05. This indicates that the proposed network (coupled with the KU-LAMP) can be utilized as a portable screening device for rapid COVID-19 screening as per the clinical standards.

**Table 5:** Clinical trials of the proposed framework in comparison with the clinician*.

Case	Sample name	Proposed network	Clinician’s recommendation	Ground truth
1	DSC00013.jpg	Positive	Positive	Positive
2	DSC00095.jpg	Negative	Negative	Negative
3	DSC00096.jpg	Negative	Negative	Negative
4	DSC00017.jpg	Positive	Positive	Positive
5	DSC00020.jpg	Positive	Positive	Positive
6	DSC00021.jpg	Positive	Positive	Positive
7	DSC00022.jpg	Positive	Positive	Positive
8	DSC00023.jpg	Positive	Positive	Positive
9	DSC00025.jpg	Positive	Positive	Positive
10	DSC00030.jpg	Positive	Positive	Positive
11	DSC00036.jpg	Positive	Positive	Positive
12	DSC00037.jpg	Positive	Positive	Positive
13	DSC00041.jpg	Positive	Positive	Positive
14	DSC00042.jpg	Positive	Positive	Positive
15	DSC00043.jpg	Positive	Positive	Positive
16	DSC00045.jpg	Positive	Positive	Positive
17	DSC00049.jpg	Positive	Positive	Positive
18	DSC00051.jpg	Positive	Positive	Positive
19	DSC00055.jpg	Positive	Positive	Positive
20	DSC00059.jpg	Negative	Positive	Positive
21	DSC00060.jpg	Positive	Positive	Positive
22	DSC00061.jpg	Positive	Positive	Positive
23	DSC00062.jpg	Positive	Positive	Positive
24	DSC00063.jpg	Positive	Positive	Positive
25	DSC00065.jpg	Positive	Positive	Positive
26	DSC00068.jpg	Positive	Positive	Positive
27	DSC00071.jpg	Positive	Positive	Positive
28	DSC00075.jpg	Positive	Positive	Positive
29	DSC00083.jpg	Positive	Positive	Positive
30	DSC00086.jpg	Positive	Positive	Positive
31	DSC00087.jpg	Positive	Positive	Positive
32	DSC00088.jpg	Positive	Positive	Positive
33	DSC00089.jpg	Positive	Positive	Positive

*These scans will be released publicly on GitHub upon paper acceptance.

**Table 6:** Pearson correlation and Cramer's $\boldsymbol{\varphi }$ coefficient between the proposed network and the clinician’s grading.

Cramer's $\boldsymbol{\varphi }$ coefficient (${{\varvec{c}}}_{{\varvec{v}}}$)	0.4122
Pearson correlation coefficient (${\varvec{r}}$)	0.8032
Statistical significance (${\varvec{p}}$)	$1.85 \mathrm{x }{10}^{-8}$

### Performance validation of KU-LAMP with clinical samples against RT-qPCR method

To determine KU-LAMP's performance and evaluate its specificity and sensitivity against the gold standard RT-qPCR method, all the available clinical samples (i.e., 319) were utilized. In parallel, the aliquots from the same samples were used to perform RT–qPCR reactions to compare both results. In the current work, three different detection assays—Allplex assay (Seegene), cobas SARS-CoV-2 assay (cobas 6800 system, Roche), and NeoPlex COVID-19 Detection Kit (GeneMatrix Inc.) —were used and RT-qPCR amplification was performed using either of the three assays for a given sample (see Supplementary Information). It is also worth mentioning that the performance validation was completed on different days using different KU-LAMP portable devices, indicating that the RT-LAMP assay was reproducible from day to day, from device to device, and from position to position on the device.

**Figure 9:**
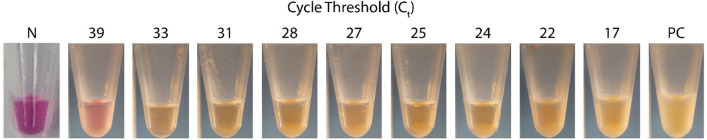
Validation results of the RT-LAMP assays performed using KU-LAMP. The photograph shows the pink to yellow/orange color change in the positive samples—with different RT qPCR-derived Ct values — at the end of the 35 min incubation at 65 °C, in comparison to the fuchsia color that is typical for the negative samples (N).

As mentioned in the preceding section, the RT-LAMP assay does not require any separate kit or instrument for RNA extraction. The incubation of the samples preceded by heat pretreatment for the lysis, both at 65° C, sets off the amplification reaction. Figure 9 shows the output of the KU-LAMP reaction output samples along with their C_t_ value (obtained from the RT qPCR test runs). As evidenced by the color change, it was possible to detect positive SARS CoV-2 cases that yielded a C_t_ as high as 39, using 2 μl of heat-treated (65° C for 10 min) nasopharyngeal samples.

**Figure 10:**
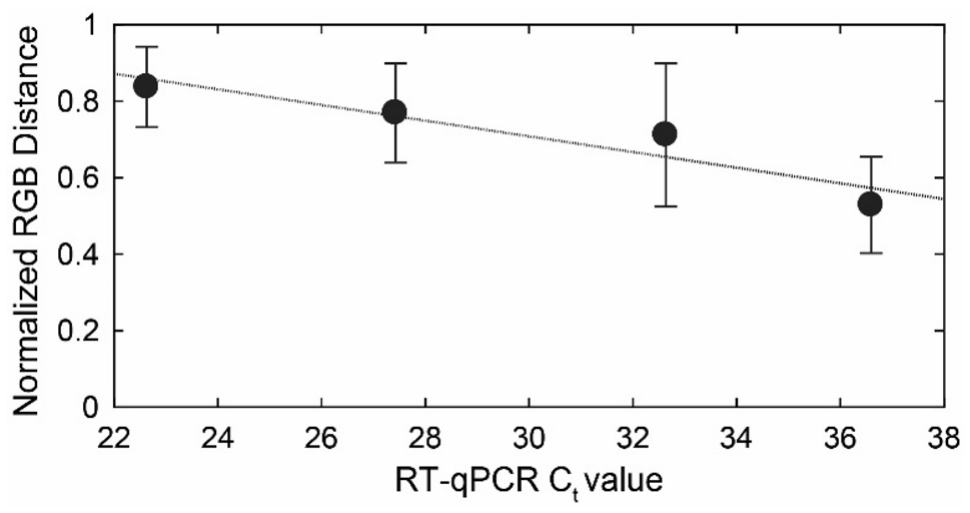
Quantification of the pink-to-yellow/orange color change in the positive samples compared to the Negative Control, as a function of the RT-qPCR cycle threshold C_t_. The quantification is performed by computing the color difference between the positive samples and the Negative Control, normalized to the difference between the Positive Control and Negative Control using the R, G, B linear dimensions defining the color space. The standard error bars represent the distribution of the normalized RGB distance for the samples categorized in four Ct bins of 0–25, 25–30, 30–35, and 35–40. (The figure is generated using MATLAB).

The dependence of the degree of pink-to-yellow color change on the C_t_ value was quantified by computing the color difference between the positive samples and Negative Control, normalized to the difference between the Positive Control and Negative Control using the R, G, B linear dimensions defining the color space: $RGB\,distance\,ratio= \frac{\sqrt{{\left({R}_{s}-{R}_{NC}\right)}^{2}+{\left({G}_{s}-{G}_{NC}\right)}^{2}+{\left({B}_{s}-{B}_{NC}\right)}^{2}}}{\sqrt{{\left({R}_{PC}-{R}_{NC}\right)}^{2}+{\left({G}_{PC}-{G}_{NC}\right)}^{2}+{\left({B}_{PC}-{B}_{NC}\right)}^{2}}}$ where the subscripts s, NC, and PC are for the sample, Negative Control, and Positive Control, respectively. All the samples were photographed under the same conditions. We categorized the RT-qPCR-positive samples by their C_t_ values into four bins (as listed in Table 7). For each bin, we calculated the average normalized RGB distance and the average C_t_, as plotted in Fig. 10. The standard deviation error bars shown in the Fig. 10. represent the distribution of the normalized RGB distance of the samples categorized in each of these C_t_ bins. The data demonstrates a correlation between the color difference and the RT qPCR-derived C_t_ value, wherein the color difference is higher for lower C_t_ values.

**Table 7:** RT-qPCR and KU-LAMP testing of 319 clinical samples categorized into the groups of C_t_ value bins.

Samples		RT-qPCR				KU-LAMP	Mismatch	KU-LAMP Specificity (%)	KU-LAMP Sensitivity (%)
		Allplex	Cobas	NeoPlex	Total				
Negative		25	25	48	98	98	0	100	–
Positive	C_t_ < 25	13	13	15	41	41	0	–	100
	C_t_ = 25–30	19	15	18	52	51	1	–	98.92
	C_t_ = 30–35	17	36	34	87	75	12	–	86.21
	C_t_ > 35	12	6	23	41	36	5	–	87.80
Total		86	95	138	319	301	18	–	94.36

The overall clinical specificity (estimated using the ratio of the number of true negatives over the number of true negatives plus the number of false positives) was calculated to be 100% since all 98 negative samples detected by the three PCR assays came out negative in KU-LAMP. Moreover, the sensitivity of KU-LAMP (the ratio of the number of true positives over the number of true positives plus the number of false negatives) for C_t_ < 30 was 98.92%, with only one (01) false-negative result out of the 93 samples. Finally, 18 samples from the 319 samples that were identified as positive by either of the RT-qPCR assays did not exhibit a significant color change to be termed as a positive result in the KU-LAMP. Hence, the overall sensitivity of the device was calculated to be 94.36%.

**Figure 11:**
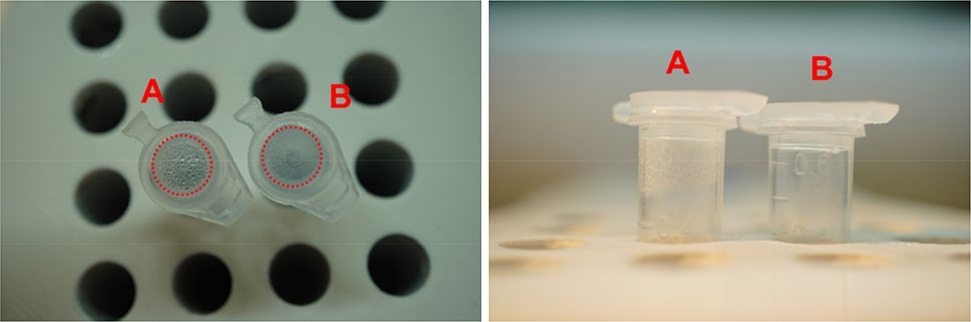
The top and side view images of the amplification in the tubes with and without the silicone oil layer. The silicone oil was not added to sample in the tube labeled (“**A**”), which shows condensation on the sidewalls and the cap. In contrast, the tube labelled as (“**B**”) contains 5 µl of silicone oil encapsulating the sample and preventing evaporation.

To investigate the effect of the silicone oil on the amplification process, the RT-LAMP was performed using two additional samples with and without the silicone oil. The results of both cases are displayed in Fig. 11. The condensation, observed on the sides and the top of the tubes without the silicone oil clearly demonstrates a loss of the sample volume. Having evaporation is undesirable in the amplification process, especially considering the tiny amount of the sample used. In contrast, the oil layer works favorably in tube B, as it encapsulates the sample completely and prevents evaporation. Moreover, with the silicone oil, we observed a sharpness in the final colors of the samples after the amplification, thus producing improved results with KU–LAMP.

Finally, KU-LAMP is developed using readily available off-the-shelf components. The cost of the KU-LAMP system is calculated to be about $150, which makes it highly affordable compared to the conventional RT–qPCR technology. Moreover, the developed testing system enables rapid diagnosis without a need for qualified personnel. These qualities offer great potential for expanding the current COVID-19 diagnostic capacity with minimal financial and training investments. Currently, KU-LAMP is designed to perform 16 simultaneous RT-LAMP reactions. This capacity is partially determined by the small size of the heating element and the overall low power consumption, consistent with the target qualities of being portable and low-voltage operational (i.e., 5 Volts). An increase in the device's capacity is possible through using smaller tubes and/or by allowing the use of a larger heating element and increased electrical power input. Nevertheless, the device is highly scalable, and the number of parallel reactions will be increased in the future prototypes by incorporating a higher power supply and integrated control circuitry for the heating modules. Furthermore, the device is not limited to the detection of SARS-CoV-2. The timers and temperature controllers can be easily reprogrammed to diagnose other pathogens.

## Conclusion

With a fast-growing number of COVID-19 cases, there is a strong and urgent need to introduce alternative diagnostic systems that can supplement the conventional COVID-19 screening based on RT-qPCR. The current work presents one such device (dubbed KU-LAMP) capable of performing multiple (16 at a time) RT-LAMP reactions for the autonomous detection of COVID-19. The accurate, rapid, portable, low power, and low-cost testing system performs the RT-LAMP process using nasopharyngeal swab specimens, without a need for the RNA extraction, and produces a colorimetric readout via deep learning in 45 min. The efficacy of the KU-LAMP is confirmed using a novel dataset containing 250 clinically validated RT-LAMP reaction samples on which the proposed system outperformed its competitors by achieving 1.45% improvements in terms of sensitivity and 3.56% improvements in terms of specificity. Also, the specificity of 0.9666 along with the sensitivity of 0.9722 (0.9892 for C_t_ < 30) on 250 RT-LAMP reaction samples suggest that KU-LAMP can be a significant addition to the other POC platforms for rapid COVID-19 screening. Moreover, we have also extensively tested the proposed KU-LAMP in clinical settings, where it was used to screen the positive and negative COVID-19 samples as compared to the expert clinicians. The results reported in Table 6 shows that the proposed framework achieved a statistically significant Pearson correlation and Cramer’s $\varphi$ coefficient of 0.8032 and 0.4122, respectively. This strong statistical association between the proposed system and clinician’s grading validates the applicability of the proposed system in clinical practice. Furthermore, it’s worth mentioning that all the current state-of-the-art methods are developed using chest X-rays and C.T. imagery, and, to the best of our knowledge, there is no deep learning driven COVID-19 screening system (other than the proposed system) which can recognize COVID-19 pathologies using RT-LAMP reactions. In addition to this, the utilization of RT-LAMP reactions for the autonomous COVID-19 is more clinically significant because of their high tolerance capacity against inhibitors, and their ability to amplify minimally processed or even raw samples (please see Table 1 for more details). Although the proposed system's design is highly scalable, the capacity of the tests per run is currently limited to the controlling area of the heating element and/or the dimensions/volume of the test tubes. However, this limitation can be addressed in the future by developing two versions of the device, where one variant would provide portability for rapid COVID-19 screening (for homes and office usage), and the other variant would provide flexibility towards conducting more tests simultaneously (within clinics and hospital). Also, due to the robustness of the proposed system, in future, it can be used for the diagnostics of other pathogens; offering the enhanced testing capabilities which could reduce the load on the healthcare system by making rapid and widespread testing available at the early stages of future outbreaks.

## Supplementary Information

Supplementary Information.
