# Supplementary material for: A deep learning-driven low-power, accurate, and portable platform for rapid detection of COVID-19 using reverse-transcription loop-mediated isothermal amplification
Source: Sci Rep. 2022 Mar 8;12:4132. doi: 10.1038/s41598-022-07954-2 (PMC8903312; doi:10.1038/s41598-022-07954-2)
Supplement: Supplementary file 1 — Supplementary Information. [file 41598_2022_7954_MOESM1_ESM.docx]

A Deep Learning-Driven Low-Power, Accurate, and Portable Platform for Rapid Detection of COVID-19 Using Reverse-Transcription Loop-Mediated Isothermal Amplification

Waqas Waheed^1^, Sueda Saylan^2,3^, Taimur Hassan^3,4^, Hussain Kannout^5^, Habiba Alsafar^5,6^, Anas Alazzam^1,2,a^
^1^Department of Mechanical Engineering, Khalifa University, Abu Dhabi UAE
^2^System on Chip Center (SOCC), Khalifa University, Abu Dhabi UAE
^3^Department of Electrical Engineering and Computer Science, Khalifa University, Abu Dhabi UAE
^4^Center for Cyber-Physical Systems (C2PS), EECS Department, Khalifa University, Abu Dhabi UAE
^5^Center for Biotechnology (BTC), Khalifa University, Abu Dhabi, UAE
^6^College of Medicine and Health Sciences, Khalifa University, Abu Dhabi, UAE
^a^Corresponding Author: [anas.alazzam@ku.ac.ae](mailto:anas.alazzam@ku.ac.ae)

## **Supplementary Information: RT-qPCR Validation of the clinical samples**

In the RT qPCR assays, the first step involved extracting the RNA prior to using the RT-qPCR assay. The extracted RNA was then aliquoted and RT qPCR was performed by using either of the three different RT-qPCR assays, namely Allplex assay (Seegene), cobas SARS-CoV-2 assay (cobas 6800 system, Roche), and NeoPlexTM COVID-19 Detection Kit (GeneMatrix Inc., Lot A107) for 40 cycles. The reaction mixture in each RT-qPCR assay was prepared by following the manufacturer’s protocol. Figure S1 depicts the schematics of the RT-qPCR process for the NeoPlexTM COVID-19 Detection Kit.


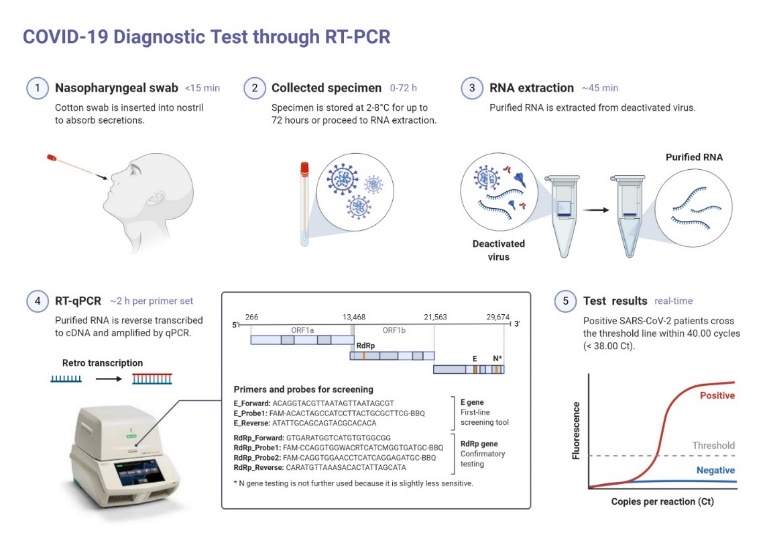


Fig. S1: Schematics of the protocol followed in RT-qPCR using NeoPlex COVID-19 Detection Kit for COVID-19 detection. Nasopharyngeal swabs were collected and were utilized either immediately or within 72 hours. The RNA in each sample was extracted using a Promega Maxwell RSC instrument (Promega Corporation, Madison, USA) with an insert extraction kit Maxwell RSC Viral TNA Lot# 110107 (Promega Corporation, Madison, USA) and the RT qPCR amplification was performed on the aliquot using NeoPlex (CE-IVD, NR05A, Lot A107) for 40 cycles on BIO-RAD CFX96 instrument (Bio-Rad Laboratories, Inc, Hercules, California, United States). The figure is generated using BioRender software.

Figure S2 shows the amplification curves of the RT–qPCR performed using NeoPlex COVID-19 detection assay; Fig. S2.a represents the positive samples, while Fig. S2.b represents the negative samples. The green–colored curves indicate that there is an amplification of the N gene either of the Positive Control sample or of the SARS-CoV-2 N gene in the patient sample; whereas, the purple-colored curves are indicative of the Internal Control (IC). In Fig. S2.a, all the green curves represent a successful amplification with each cycle crossing the fluorescence threshold at a different value in the range of 0 40 cycles. This value is called the cycle threshold (C_t_) level and is an indication of the viral load in a sample. The lower the C_t_ value, the greater the amount of nucleic acid in the sample and vice versa. Furthermore, all the internal control samples (purple curves) are also amplified, which indicates that the extraction step and PCR amplifications are valid for all the positive samples. Figure S2.b shows the amplification curves for all the negative samples used in the current study. It can be seen that only the positive control sample (the green curve) showed amplification, while the negative samples did not show any amplification. Again, the PCR process was validated by the fact that all the internal control samples (purple curves) were amplified, as shown in S2.a and S2.b.


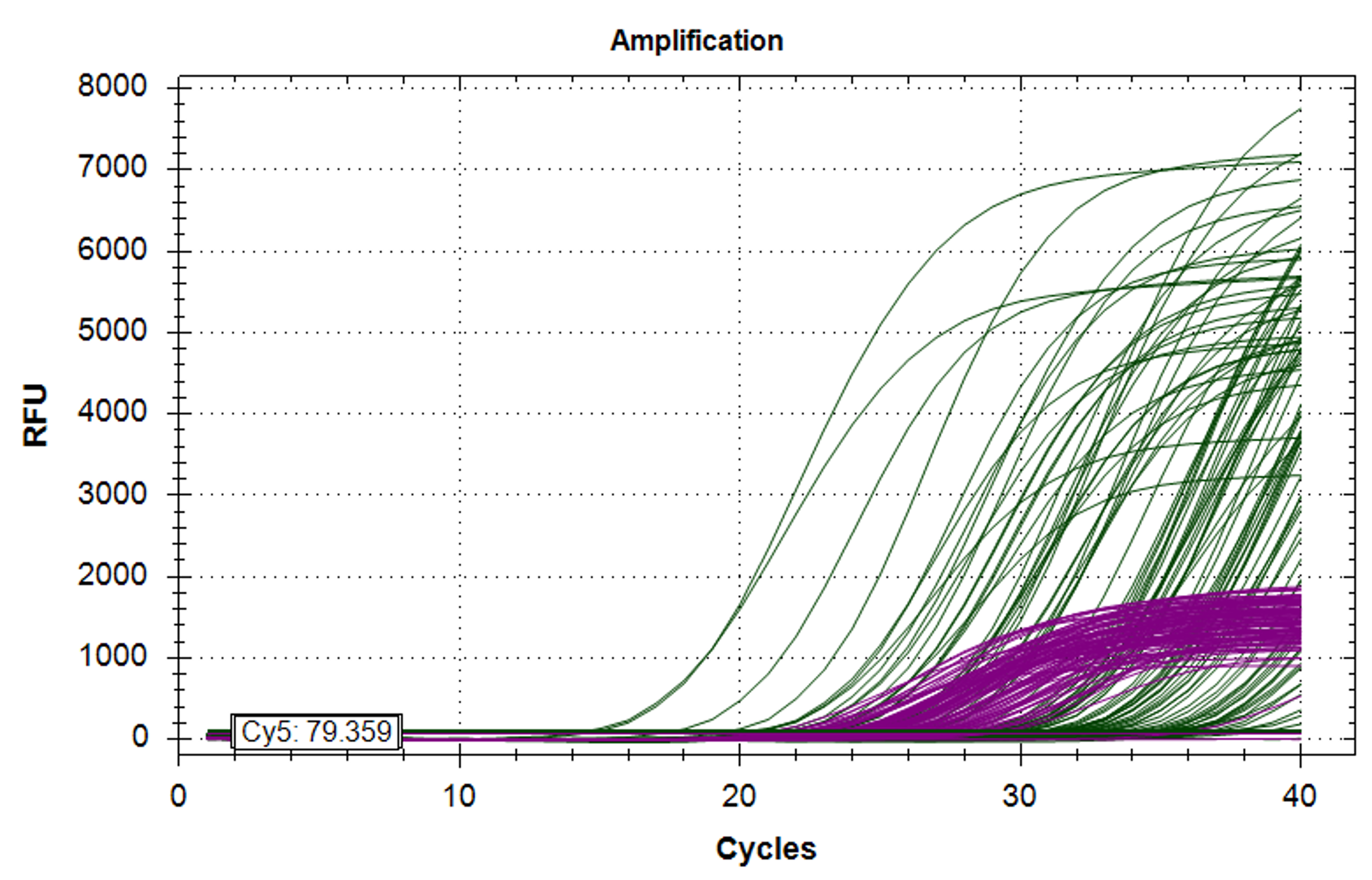


(a)


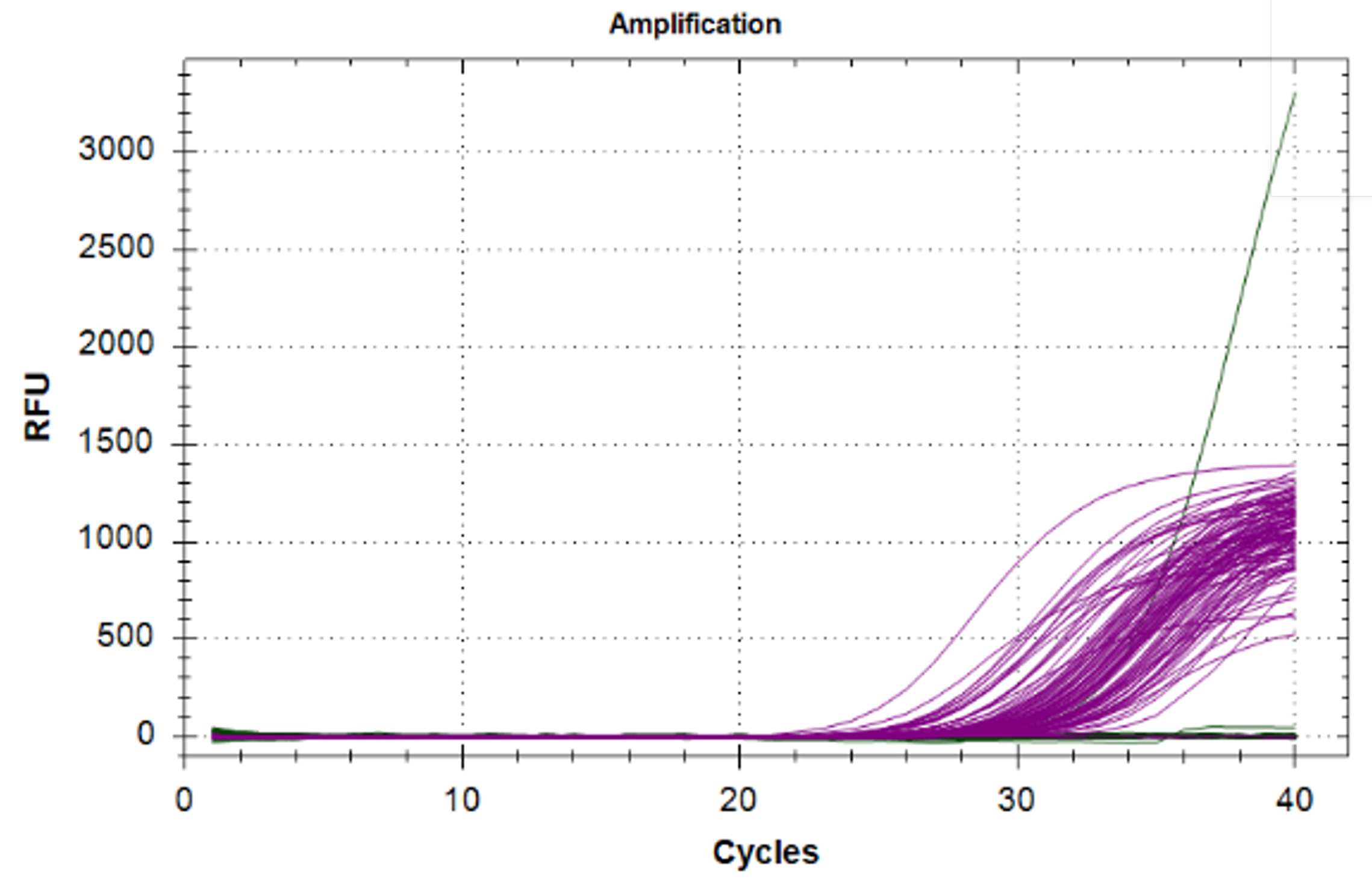


(b)

Fig. S2: The amplification curves for (S2.a) positive samples and (S2.b) negative samples. The green curves represent the N gene, while the purple curves represent the Internal Control (IC). The curves are generated using the software provided with PCR Machine cfx96.
